# The cytoprotective role of GM1 ganglioside in Huntington disease cells

**DOI:** 10.1007/s11033-022-07830-2

**Published:** 2022-09-30

**Authors:** Hannah S. Hart, Madeline A. Valentin, Stephanie Toering Peters, Susan W. Holler, Hongmin Wang, Aaron F. Harmon, Larry D. Holler

**Affiliations:** 1 GlycoScience Research, Brookings, SD USA; 2 Department of Biomedical Engineering, University of South Dakota, Sioux Falls, SD USA; 3 Department of Biology, Wartburg College, Waverly, IA USA; 4 Division of Basic Biomedical Sciences and Center for Brain and Behavior Research, Sanford School of Medicine, University of South Dakota, Vermillion, SD USA

**Keywords:** GM1 ganglioside, Huntington’s disease, Juvenile Huntington’s disease, Mutant huntingtin, Polyglutamine repeats, Cytoprotection

## Abstract

**Background:**

Huntington disease (HD) is a neurodegenerative disease where a genetic mutation leads to excessive polyglutamine (Q) repeats in the huntingtin protein. The polyglutamine repeats create toxic plaques when the protein is cleaved, leading to neuron death. The glycolipid GM1 ganglioside (GM1) has been shown to be neuroprotective in HD models, as it prevents the cleavage of the mutant huntingtin protein by phosphorylation of serine 13 and 16. Previous studies have tested GM1 in both adult-onset and juvenile-onset HD models, but this study set out to investigate whether GM1 mediated cytoprotection is influenced by the length of polyglutamine repeats.

**Method and result:**

This study utilized cell culture to analyze the effect of GM1 on cell viability, directly comparing the response between cells with adult-onset HD and juvenile-onset HD. HEK293 cells expressing either wild-type huntingtin (Htt) (19Q) exon 1, adult-onset HD mutant Htt exon 1 (55Q), or Juvenile HD mutant Htt exon 1 (94Q) were assessed for cell viability using the WST-1 assay. Our results suggested moderate doses of GM1 increased cell viability for all cell lines when compared to untreated cells. When comparing HEK293 55Q and 94Q cells, there was no difference in cell viability within each dose of GM1.

**Conclusion:**

These data suggest cellular responses to GM1 are independent of polyglutamine repeats in HD cells and provide insight on GM1’s application as a therapeutic agent for HD and other diseases.

## Introduction

Huntington disease (HD) is an autosomal dominant, neurodegenerative disease caused by a mutation which results in an expanded polyglutamine (Q) tract near the N-terminus in the huntingtin gene. When cleaved, the fragments are prone to form toxic aggregates. Fewer than 26 repeats are considered normal, but HD symptoms present when polyglutamine repeats exceed 36. As repeats increase beyond 36, age of onset decreases, and severity of the disease intensifies. Juvenile HD occurs as repeats exceed 60. Neurodegredation is most obvious in the basal ganglia, which includes the striatum and globus pallidus, and is eventually evident throughout the cortex and cerebellum [1].

GM1 ganglioside (GM1), a glycolipid, has been shown to have reduced levels of expression in models of HD, lowering the threshold for cell death [2, 3]. GM1 is highly concentrated in cell membranes, where it facilitates interactions between cells, membrane proteins, and supporting cells [4]. One of GM1’s most significant roles is in neuroprotection and repair [3]. In addition, GM1 influences the proteasome system’s ability to break down protein fragments, and if deficient in HD, helps to explain the accumulating toxicity of mHtt [5].

GM1 can be introduced in HD models to promote pro-survival pathways. GM1 activates the AKT kinase [6], resulting in phosphorylation at serine 13 and serine 16 in the mHtt protein [3, 7, 8]. Phosphorylation prevents cleavage of mHtt and the formation of plaques. Preventing the cleavage of mHtt lowers mHtt toxicity and preserves the normal huntingtin protein’s role [9]. In addition, GM1 has been shown to restore motor function in symptomatic mice [10]. Following treatment with GM1 in a mouse model, anxiety, depression, and memory were restored to normal levels [11].

We sought to determine if GM1’s influence in HD would be affected by the increased polyglutamine repeats associated with juvenile-onset HD. Therefore, we investigated if a protective effect on cell viability could be obtained through GM1 treatment of three human kidney cell lines (HEK293) containing increasing numbers of polyglutamine repeats of either 19Q, 55Q, or 94Q. These cells were selected to simulate cellular-level disease of normal, adult-onset, and juvenile-onset HD. The GM1 utilized in this study was of ovine source, purchased from Avanti Polar Lipids.

## Materials and method

### Cell lines and treatment groups

HEK293(CRL-1573), human embryonic kidney epithelial cell line, obtained from the ATCC, was previously modified to stably express Htt exon 1 containing specific numbers of polyglutamine repeats. The modifications were verified in our previous studies [12]. The first line of HEK293 cells expressed 19 polyglutamine repeats (19Q), acting as the normal model. The next cell line expressed 55 repeats (55Q), indicative of adult-onset HD. The final cell line expressed 94 repeats (94Q) and represents the more severe juvenile-onset HD. Cells were maintained in a medium containing DMEM (high-glucose), 10% FBS, and penicillin/streptomycin and were stored in an incubator at 37 °C with 5% CO_2_ saturation [12].

### GM1 ganglioside

GM1 was obtained from Avanti Polar Lipids, isolated from ovine brain (Cat#:860065). For application to cell lines, GM1 was dissolved into Dulbecco’s phosphate buffered saline (DPBS). The stock GM1 solution was added directly to the plates or additional media to achieve the desired concentrations.

### Measuring cell viability with treatment of GM1 ganglioside

Each cell line was plated separately on a 96-well plate, and cells were grown to 90–100% confluence in an incubator at 37 °C with 5% CO_2_ saturation. GM1 was applied to the confluent cells for 24 h. A subset of the wells was treated with DPBS only (0 μg/ml GM1). Equal numbers of wells were then treated with GM1 in concentrations of 0.002, 0.02, 0.2, 2, 20, and 200 μg/ml GM1. After 24 h, the WST-1 Quick Cell Proliferation Colorimetric Assay Kit (Biovision#K301) was utilized to measure cell viability following the manufacturer’s protocol. A subset of the wells on each plate acted as a control and were treated with DPBS only. Equal numbers of wells were then treated with the experimental concentrations of GM1 with concentrations of 0.002, 0.02, 0.2, 2, 20, and 200 μg/ml GM1 [3, 10]. The layout of dosages on each plate were randomized and researchers who analyzed the data were blinded. On a 2 predictor Tukey HSD power test with a power level of 0.7 and a probability level of 0.1 (α), it was found a sample size of 18 would be sufficient to compare any two sample treatment combinations. The minimum sample size achieved for comparison was 11 for HEK293 94Q cells because of difficulty growing the diseased cells, but the average comparison was 18 for 19Q and 55Q cells.

### Statistical analysis

**Fig. 1:**
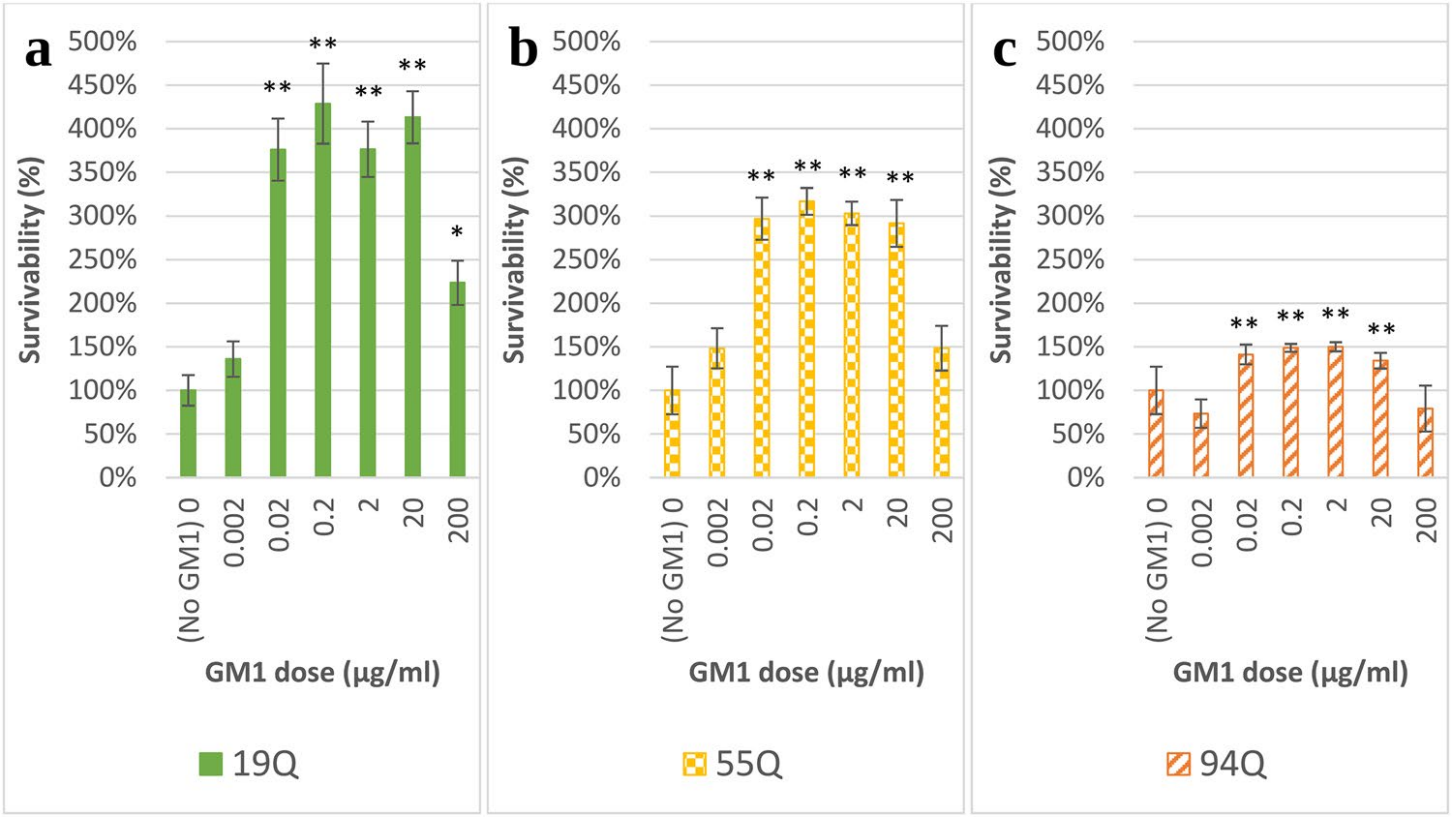
Moderate doses of GM1 increase HEK293 cell viability as evaluated by the WST-1 assay. The average viability for cells exposed to GM1 is shown for each cell line, separately. **a** 19Q, **b** 55Q, **c** 94Q. Drug doses increase from 0 to 200 μg/ml of GM1 left to right. Error bars represent the standard error of the group mean. Moderate dosages of GM1 significantly increased cell viability compared to untreated cells with 0 μg/ml of GM1 as indicated by ANOVA and Tukey HSD tests. A sample was defined as one well within the 96-well plate. The 19Q cell line had 12 samples within the control and 18 samples within each dose of GM1 for a total of 120 samples. The 55Q cell line had 6 samples within the control and 9 samples within each dose of GM1 for a total of 60 samples. The 94Q cell line had 6 samples within the control and 5 samples within each dose of GM1 for a total of 36 samples. *Tukey HSD tests indicate the group is significantly different compared to 0 μg/ml of GM1 where p < 0.05. **Tukey HSD tests indicates the group is significantly different compared to 0 μg/ml of GM1 where p < 0.01

The average viability of cells in response to GM1 was first compared *within* each cell line (Fig. 1). Data were normally distributed. Within each cell line, ANOVA was used to compare the seven doses of GM1. Next, Tukey HSD post hoc tests compared each dosage of GM1 against the untreated cells (0 μg/ml GM1) for the respective cell line.

**Fig. 2:**
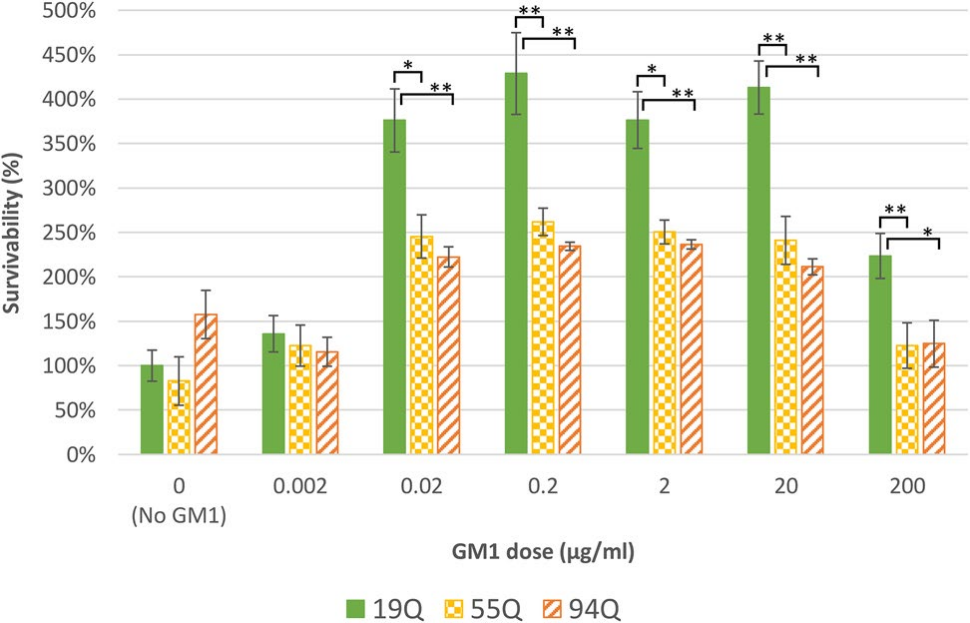
Cell viability after treatment with GM1 is similar between adult-onset and juvenile-onset HD cells. The average viability for cells exposed to GM1 is shown for all cell lines. The data have been normalized to directly compare the response to GM1 between cell lines. To normalize, the mean survivability of untreated 19Q cells was set as 100% survival, and the averages of other cell lines and treatments were divided by the average survivability of untreated 19Q cells (0 μg/ml GM1). Drug doses increase from 0 to 200 μg/ml GM1. Error bars represent the standard error of the group mean. A sample was defined as one well within the 96-well plate. The 19Q cell line had 12 samples within the control and 18 samples within each dose of GM1 for a total of 120 samples. The 55Q cell line had 6 samples within the control and 9 samples within each dose of GM1 for a total of 60 samples. The 94Q cell line had 6 samples within the control and 5 samples within each dose of GM1 for a total of 36 samples. *HSD tests indicate there is a significant difference between the two cell lines for the specific GM1 dose where p < 0.05. **Tukey HSD tests indicate there is a significant difference between the two cell lines for the specific GM1 dose where p < 0.01.  All data were considered significant with p < 0.05

The average viability of cells in response to GM1 was then compared *between* cell lines (Fig. 2). To allow for direct comparison between cell lines at each dose of GM1, the data were normalized. To normalize, each data point for the 19Q, 55Q, and 94Q cell lines were divided by the average survivability of the untreated 19Q cells (0 μg/ml GM1). The average survivability for each cell line’s control dosage were therefore normalized to each other and each data point scaled proportionally. The normalized data were normally distributed. ANOVA tests were first used to compare the three cell lines at each dose of GM1. Next, Tukey HSD post hoc tests compared each cell line’s viability to one another within that dose of GM1.

## Results

### Moderate doses of GM1 ganglioside increase viability in HEK293 HD cells

HEK293 19Q cells had increased cell viability compared to untreated cells with 0 μg/ml GM1 when treated with 0.02, 0.2, 2, 20 and 200 μg/ml GM1 (Fig. 1a). A one way ANOVA indicated a significant effect of GM1 dosage on cell viability for the seven dosages of GM1 [F_6_ = 27.21, p = 1.11e−16]. Post hoc comparisons utilizing Tukey HSD tests indicated the average viability for 19Q cells exposed to 0.02 μg/ml [Q_29_ = 10.41, p = 6.11e−08], 0.2 μg/ml [Q_29_ = 10.48, p = 4.68e−08], 2 μg/ml [Q_29_ = 10.43, p = 5.79e−08], 20 μg/ml [Q_29_ = 11.81, p = 6.33e−10], and 200 μg/ml [Q_29_ = 4.66, p = 0.022] GM1 were significantly higher than untreated cells exposed to 0 μg/ml GM1. However, viability for cells exposed to 0.002 μg/ml GM1 were not significantly different than untreated cells exposed to 0 μg/ml GM1.

HEK293 55Q cells had increased cell viability compared to untreated cells with 0 μg/ml GM1 when treated with 0.02, 0.2, 2, and 20 μg/ml GM1 (Fig. 1b). A one way ANOVA indicated a significant effect of GM1 dosage on cell viability for the seven dosages of GM1 [F_6_ = 14.42, p = 1.15e−09]. Post hoc comparisons utilizing Tukey HSD tests indicated the average viability for 55Q cells exposed to 0.02 μg/ml [Q_14_ = 7.68, p = 0.00046], 0.2 μg/ml [Q_14_ = 8.45, p = 8.29e−05], 2 μg/ml [Q_14_ = 7.91, p = 0.00028], and 20 μg/ml [Q_14_ = 7.47, p = 0.0073] GM1 were significantly higher than untreated cells exposed to 0 μg/ml GM1. However, viability for cells exposed to 0.002 μg/ml and 200 μg/ml GM1 were not significantly different than untreated cells exposed to 0 μg/ml GM1.

HEK293 94Q cells had increased cell viability compared to untreated cells with 0 μg/ml GM1 when treated with 0.02, 0.2, 2, and 20 μg/ml (Fig. 1c). A one way ANOVA indicated a significant effect of GM1 dosage on cell viability for the seven dosages of GM1 [F_6_ = 35.48, p = 4.42e−12]. Post hoc comparisons utilizing Tukey HSD tests indicated the average viability for 94Q cells exposed to 0.02 μg/ml [Q_10_ = 11.58, p = 1.90e−06], 0.2 μg/ml [Q_10_ = 12.47, p = 4.39e−07], 2 μg/ml [Q_10_ = 12.36, p = 5.29e−07], and 20 μg/ml [Q_10_ = 10.08, p = 2.35e−05] GM1 were significantly higher than untreated cells exposed to 0 μg/ml GM1. However, viability for cells exposed to 0.002 μg/ml and 200 μg/ml GM1 were not significantly different than untreated cells exposed to 0 μg/ml GM1.

### The response to GM1 between adult-onset and Juvenile-onset HEK293 cells is similar

There were no differences in cell viability between HEK293 55Q and 94Q cells for dosages of 0, 0.002, 0.02, 0.2, 2, 20 or 200 μg/ml GM1 (Fig. 2). HEK293 19Q cells had higher viability than 55Q cells for doses of 0.02, 0.2, 2, 20 and 200 μg/ml GM1 (Fig. 2). HEK293 19Q cells had higher viability than 94Q cells for doses of 0.2, 20, and 200 μg/ml GM1 (Fig. 2).

One way ANOVA tests showed a significant effect of cell line on cell viability at doses of 0.02, 0.2, 2, 20 and 200 μg/ml GM1.

At the dosage of 0 μg/ml GM1, ANOVA indicated no significant difference in cell viability across HEK293 19Q, 55Q, and 94Q cells.

At the dosage of 0.002 μg/ml GM1, ANOVA indicated no significant difference in cell viability across HEK293 19Q, 55Q, and 94Q cells.

At the dosage of 0.02 μg/ml GM1, ANOVA indicated a significant difference in cell viability across HEK293 19Q, 55Q, and 94Q cells [F_2_ = 7.10, p = 0.027]. Post hoc Tukey HSD tests indicated 19Q cells had higher viability than 55Q cells at 0.02 μg/ml GM1 [Q_26_ = 3.97, p = 0.022]. 19Q cells also had higher viability than 94Q cell lines [Q_22_ = 4.67, p = 0.0063]. No significant difference in viability was identified between 55 and 94Q cell lines at 0.02 μg/ml GM1.

At the dosage of 0.2 μg/ml GM1, ANOVA indicated a significant difference in cell viability across HEK293 19Q, 55Q, and 94Q cells [F_2_ = 9.41, p = 0.0010]. Post hoc Tukey HSD tests indicated 19Q cells had higher viability than 55Q cells at 0.2 μg/ml GM1 [Q_26_ = 4.87, p = 0.0058]. 19Q cells also had higher viability than 94Q cell lines [Q_22_ = 5.67, p = 0.0014]. No significant difference in viability was identified between between 55 and 94Q cell lines at 0.2 μg/ml GM1.

At the dosage of 2 μg/ml GM1, ANOVA indicated a significant difference in cell viability across HEK293 19Q, 55Q, and 94Q cells [F_2_ = 7.65, p = 0.0019]. Post hoc Tukey HSD tests indicated 19Q cells had higher viability than 55Q cells at 2 μg/ml GM1 [Q_26_ = 3.02, p = 0.018]. 19Q cells also had higher viability than 94Q cell lines [Q_22_ = 3.35, p = 0.0079]. No significant difference in viability was identified between 55 and 94Q cell lines at 2 μg/ml GM1.

At the dosage of 20 μg/ml GM1, ANOVA indicated a significant difference in cell viability across HEK293 19Q, 55Q, and 94Q cells [F_2_ = 15.90, p = 1.46e−05]. Post hoc Tukey HSD tests indicated 19Q cells had higher viability than 55Q cells [Q_26_ = 5.96, p = 0.0010] and between 19 and 94Q cell lines [Q_22_ = 6.99, p = 0.0010] at 20 μg/ml GM1. No significant difference in viability was identified between 55 and 94Q cell lines at 20 μg/ml GM1.

At the dosage of 200 μg/ml GM1, ANOVA indicated a significant difference in cell viability across HEK293 19Q, 55Q, and 94Q cells [F_2_ = 7.25, p = 0.0025]. Post hoc Tukey HSD tests indicated 19Q cells had higher viability than 55Q cells [Q_26_ = 4.44, p = 0.0096] and between 19 and 94Q cell lines [Q_22_ = 4.35, p = 0.011] at 200 μg/ml GM1. No significant difference in viability was identified between 55 and 94Q cell lines at 200 μg/ml GM1.

## Discussion

The primary aim of this study was to compare the response to GM1 between cells representing adult-onset HD and juvenile-onset HD. This study specifically compared whether increasing numbers of polyglutamine repeats in HD cells would affect the required dosage at which GM1 could be therapeutic. As the number of polyglutamine repeats is correlated with disease severity and inversely correlated with onset age of HD [1], it is pertinent to consider if modified dosages of GM1 would be required in more severe cases. However, this study never showed a significant difference in cell viability between HEK293 55Q and 94Q cells within each dose of GM1. These data suggest, between adult-onset HD and juvenile-onset HD, the response to GM1 is not dependent on polyglutamine repeats. There were differences in viability between the 19Q cells and both the 55Q and 94Q cells at doses above 0.02 μg/mL GM1.

Research investigating how the number of polyglutamine repeats affect mHtt cellular disfunction is ongoing. It is agreed that a greater number of polyglutamine repeats causes amyloid like aggregates, composed primarily of β-sheets, to form more rapidly [13]. While folding and fibrilization occur faster as repeats increase, the order of progression is uniform and independent of the number of repeats [14]. All experiments in this study measured cell viability 24 h after GM1 addition. This exceeded the 12-h mark at which full mHtt aggregate formation and cytotoxic effects, characteristic of aggregate buildup, manifest in cells of multiple polyglutamate repeat length, according to Sahoo et al. [14]. Therefore, we postulate the cellular response to GM1 was similar in HEK 55Q and 94Q cells, as GM1 would have equally toxic levels of protein aggregates, reminiscent of HD, to clear in both cell lines.

This study found moderate doses of GM1 significantly increased cell viability for all cell lines. These data suggest dosages between 0.02 and 20 μg/mL GM1 most effectively increase viability as each cell line showed a significant increase compared to untreated cells, regardless of the number of polyglutamine repeats. Additionally, at the highest dose of GM1, 200 μg/mL GM1, viability was never significantly different than untreated cells exposed to 0 μg/mL GM1, which is not surprising, since an over accumulation of GM1 can have severe pathology as seen in patients with GM1 gangliosidosis.

An impressive body of research exists detailing GM1’s therapeutic effects on cellular, behavioral, and systemic levels [10, 11, 15, 16, 17]. Given the depth of literature describing GM1’s promotion of the AKT pathway [3, 7, 8], we theorized the same mechanism is at work here to increase cell viability in diseased HEK293 cells compared to untreated cells.

The HEK293 19Q cell line had increased viability with GM1 administration compared to untreated cells. This can be partially explained by the cytoprotective effect of GM1 [3, 5]. GM1’s function is synonymous with neurotrophin functionality [5, 18]. GM1 is also essential for TrkA activity [19], BDNF release [20], and anti-inflammation in supporting cells [21]. GM1’s importance in the body explains, in part, the deleterious effects seen in HD if the ganglioside is deficient [22], but it may also explain why the cell line expressing no HD (19Q) experienced increased survivability with GM1.

Because this study utilized kidney cells, it is relevant to wonder how the findings might translate to neurons. Previously, HD mouse brains injected with GM1 showed increased brain, striatal, and corpus collosum volume compared to untreated HD mouse brains [11]. The astrocyte-supporting, glial fibrillary acidic protein (GFAP) was also restored to wild type levels with GM1 administration [11]. Still, replicating this in-vitro study with neurons is warranted.

This study suggests the severity of HD does not influence the response to GM1. Additionally, this study supports the cytoprotective role of GM1. Mechanisms directly comparing adult-onset HD to juvenile-onset HD still require further investigation. GM1’s ability to trigger phosphorylation at serine 13 and 16 to clear mHtt, the subsequent improvements to HD symptoms in mouse models, and its success in clinical trials outside of HD [23, 24, 25] continue to affirm GM1 ganglioside as a potential therapeutic in Huntington Disease.

## Data Availability

The datasets generated during and analyzed during the current study are available from the corresponding author on reasonable request.
